# Promoter methylation of *Wnt5a* is associated with microsatellite instability and BRAF V600E mutation in two large populations of colorectal cancer patients

**DOI:** 10.1038/bjc.2011.165

**Published:** 2011-05-17

**Authors:** J B Rawson, M Mrkonjic, D Daftary, E Dicks, D D Buchanan, H B Younghusband, P S Parfrey, J P Young, A Pollett, R C Green, S Gallinger, J R McLaughlin, J A Knight, B Bapat

**Affiliations:** 1 Department of Laboratory Medicine and Pathobiology, University of Toronto, Toronto, Ontario, Canada M5S 1A1; 2 Samuel Lunenfeld Research Institute, Mount Sinai Hospital, 60 Murray Street, L6-304B, Box 30, Toronto, Ontario, Canada M5G 1X5; 3 Ontario Familial Colorectal Cancer Registry, Cancer Care Ontario, Toronto, Ontario, Canada M5G 2L7; 4 Faculty of Medicine, Memorial University, St John's, Newfoundland, Canada A1C 5S7; 5 Familial Cancer Laboratory, Queensland Institute of Medical Research, Herston, Queensland 4006, Australia; 6 Department of Pathology and Laboratory Medicine, Mount Sinai Hospital, Toronto, Ontario, Canada M5G 1X5; 7 Department of Surgery, University of Toronto, Toronto, Ontario, Canada M5S 1A1; 8 Dalla Lana School of Public Health, University of Toronto, Toronto, Ontario, Canada M5S 2S1

**Keywords:** colorectal cancer, methylation, Wnt, microsatellite instability

## Abstract

**Background:**

In colorectal cancer (CRC), tumour microsatellite instability (MSI) status and CpG island methylator phenotype (CIMP) status are indicators of patient outcome, but the molecular events that give rise to these outcomes remain largely unknown. Wnt5a is a critical regulator of non-canonical Wnt activity and promoter hypermethylation of this gene has emerging prognostic roles in CRC; however the frequency and prognostic significance of this epigenetic event have not been explored in the context of colorectal tumour subtype. Consequently, we investigated the frequency and prognostic significance of *Wnt5a* methylation in a large cohort of MSI-stratified CRCs.

**Methods:**

Methylation was quantified in a large cohort of 1232 colorectal carcinomas from two clinically distinct populations from Canada. Associations were examined between methylation status and clinicopathlogical features, including tumour MSI status, BRAF V600E mutation, and patient survival.

**Results:**

In Ontario, *Wnt5a* methylation was strongly associated with MSI tumours after adjustment for age, sex, and tumour location (odds ratio (OR)=4.2, 95% confidence interval (CI)=2.4–7.4, *P*<10^−6^) and with BRAF V600E mutation, a marker of CIMP (OR=12.3, 95% CI=6.9–21.7, *P*<10^−17^), but was not associated with patient survival. Concordant results were obtained in Newfoundland.

**Conclusion:**

Methylation of *Wnt5a* is associated with distinct tumour subtypes, strengthening the evidence of an epigenetic-mediated Wnt bias in CRC.

Wnt genes encode a large family of secreted glycoproteins with widespread roles in development and carcinogenesis. These proteins have classically been divided into canonical or non-canonical family members based on their ability to activate these distinct arms of the Wnt signalling network. While canonical Wnt ligands promote cell growth and proliferation by controlling cytoplasmic degradation and subsequently nuclear accumulation of *β*-catenin. Non-canonical Wnt ligands govern cell motility and polarity through the Wnt/calcium and/or planar cell polarity pathways, which act through a variety of intracellular mediators including calmodulin-dependent protein kinase II, protein kinase C, and c-Jun N-terminal kinase. Aberrant hyperactivation of the canonical Wnt pathway is a critical tumour-initiating event in the majority of colorectal cancers (CRCs); however, the status of non-canonical Wnt activity in cancer and its roles in carcinogenesis are poorly understood. These functions have mainly been examined through Wnt5a, the quintessential non-canonical Wnt ligand.

Wnt5a is ubiquitously expressed in normal tissues (Ying *et al*, 2008), but is often dysregulated in tumours (Wang *et al*, 2007, 2010) and has been controversially implicated in both tumour suppression and oncogenesis in addition to being recognised asa marker of both favourable and poor outcome in primary cancer. Studies supporting the former have demonstrated that *Wnt5a* inhibits invasion and proliferation in thyroid cancer (Kremenevskaja *et al*, 2005), low expression or loss of expression is prognostically unfavourable in breast cancer (Jonsson *et al*, 2002; Leris *et al*, 2005), and hemizygous mice develop lymphomas (Liang *et al*, 2003). On the other hand, oncogenic roles have been identified in prostate cancer, where Wnt5a promotes cell motility (Wang *et al*, 2010; Yamamoto *et al*, 2010), melanoma, where expression associates with tumour invasion (Weeraratna *et al*, 2002), and hepatocellular carcinoma, where expression is associated with poorly differentiated and invasive cell lines (Yuzugullu *et al*, 2009). In CRC, Wnt5a expression has been associated with favourable outcome in early stage tumours (Dejmek *et al*, 2005), while loss of Wnt5a expression is a hallmark of CRC-derived liver metastases (Ki *et al*, 2007). Collectively, these seemingly conflicting findings underscore the poorly understood functional and prognostic roles of Wnt5a in cancer.

Recent studies in leukaemia (Martin *et al*, 2010) and CRC (Ying *et al*, 2008; Ahn *et al*, 2011) have shown that a major mechanism for *Wnt5a* downregulation is promoter hypermethylation. Aberrant promoter hypermethylation is a hallmark of CRC that has a pivotal role in defining tumour subtypes; somatic methylation of the *MLH1* promoter causes the majority of sporadic CRC tumours with microsatellite instability (MSI), and widespread CpG island methylation defines the CpG island methylator phenotype (CIMP). Importantly, these methylation-dependent subtypes stratify disease outcome; MSI tumours are clinically favourable, but respond poorly to 5-fluorouracil-based chemotherapy (Ribic *et al*, 2003; Carethers *et al*, 2004), whereas CIMP tumours have complex associations with patient outcome that may depend on MSI and/or BRAF V600E status (Kim *et al*, 2009; Ogino *et al*, 2009; Dahlin *et al*, 2010). Despite the importance of these epigenetic modifications in defining these CRC subtypes, the specific methylation events that contribute to subtype-specific outcome has been poorly characterised.

Our previous study suggested that microsatellite stable tumours may exhibit preferential gain of non-canonical Wnt signalling through methylation of Wnt antagonists (Rawson *et al*, 2011). Given the importance of Wnt5a in mediating non-canonical Wnt activity and its emerging role in carcinogenesis and prognosis, we investigated the promoter methylation status of this gene in a large cohort of MSI-stratified CRCs from two distinct Canadian populations. We examined associations to patient clinicopathological features focusing on tumour subtype and patient survival in hopes that these relationships would provide further insight into subtype-specific epigenetic mediation of non-canonical Wnt signalling and would help define the prognostic role of *Wnt5a* methylation in CRC.

## Materials And Methods

### Study participants

Probands recruited into this study were cases of primary colorectal carcinoma accrued through the population-based Ontario Familial Colorectal Cancer Registry (OFCCR) and Newfoundland Familial Colorectal Cancer Registry (NFCCR). Patient accrual, data collection, and biospecimen collection procedures at the OFCCR have been previously detailed (Newcomb *et al*, 2007). Briefly, Ontario residents diagnosed with pathology-confirmed CRC between the ages of 20 and 74 years from 1997 to 2000 were eligible for recruitment. Clinical data were abstracted from consenting patients through several self-administered questionnaires and cancer-related medical records. Pedigrees were constructed from family history data and used to assess familial risk status according to Amsterdam status and other criteria (Raptis *et al*, 2007). Recruitment was preferential for cases with positive familial risk. Apparent cases of familial adenomatous polyposis were excluded. A total of 1004 probands with blood and/or tissue biospecimens were obtained at the time of study accrual. Due to the high prevalence of self-reported Caucasians (92.5%) and to limit the possible effects of population stratification, we excluded non-white patients and those with unknown or mixed ethnic background. Of the remaining 921 probands, we were able to obtain 600 tumour samples for methylation analysis, representing 561 of these probands. Several probands were subsequently excluded during methylation analysis due to poor quality DNA (see section MethyLight), leading to a final cohort of 545 Ontario cases. Annual follow-up was available for these probands to assess vital status and recurrence.

Case selection at NFCCR was similar to that of OFCCR except for a slightly later recruitment period (1999–2003) and the inclusion of all incident CRC patients irrespective of family history (Green *et al*, 2007). In addition, for deceased patients, NFCCR accepted proxy consent from living family members, which led to more frequent inclusion of late stage patients. A total of 747 probands were recruited by NFCCR, of which 721 probands were white (northern European origin) and had tumour samples available. Molecular analysis was successfully performed on 687 cases after removal of poor quality samples. An overview of patient clinicopathological features is shown in Table 1.

### Molecular analysis

Tumour and matched normal tissues were collected from surgical specimens and embedded in paraffin. DNA was extracted (after microdissection for Ontario patients, >70% cellularity for tumours) and used previously to analyze MSI status, *MLH1* methylation levels, and BRAF V600E mutational status.

MSI testing was performed using National Cancer Institute guidelines, as previously described (Raptis *et al*, 2007). Briefly, five or more markers were analysed from a panel consisting of: ACTC, BAT-25, BAT-26, BAT-40, BAT-34C4, D10S197, D18S55, D2S123, D5S346, and MYC-L. Microsatellite instability status was defined by the number of positive markers as follows: MSI high (⩾30% unstable markers), MSI low (1–29% unstable markers), or microsatellite stable (0% unstable markers). Newfoundland tumours that were initially characterised as MSI low were screened for a second set of markers: BAT-40, D7S519, D17S787, D18S58, and D20S100 (Woods *et al*, 2005). Due to the limited frequency of MSI low in our population and in conjunction with the classical definition of MSI status, tumours initially classified as MSI low or microsatellite stable were combined and classified as ‘MSS’. Microsatellite instability-H tumours were therefore denoted as ‘MSI’. As previously identified (Green *et al*, 2009), the frequency of MSI tumours in Newfoundland was relatively low (9.9%), which may be due, in part, to the low frequency of germline mismatch repair mutations (Woods *et al*, 2005).

Status of *MLH1* methylation was assessed by quantitative MethyLight assay as previously described (Mrkonjic *et al*, 2010). Positive methylation was defined as per cent methylated reference (PMR)>10. A large proportion of tumours in Newfoundland (37.6%) were not tested due to unavailability of tumour tissue.

The somatic c.1799T>A gene mutation (p. V600E) in the *BRAF* gene was analysed by allelic discrimination assay, as previously described in Ontario (Rawson *et al*, 2011) and in Newfoundland (Loughrey *et al*, 2007). In Ontario, 10% of samples were validated with 100% concordance. In Newfoundland, all samples with positive methylation were validated by direct sequencing.

Immunohistochemical staining of mismatch repair proteins MLH1, MSH2, MSH6, and PMS2 was performed by a trained pathologist as previously described (Lindor *et al*, 2002). Staining was classified as present, absent, or inconclusive. Tumours with absent staining in any of these proteins were defined as mismatch repair deficient.

### MethyLight

Methylation analysis was performed on matched tumour and normal tissue using semi-quantitative MethyLight assay, a Taqman-based technique that assesses per cent DNA methylation at a single gene locus per reaction (Eads *et al*, 2000). Sodium bisulphite treatment was performed on 50 ng of sample DNA using EZ DNA Methylation Gold kit (Zymo Research Corp., Orange, CA, USA) according to the manufacturer's protocol. Modified DNA was stored at −20°C until use. Primers and probes were designed to amplify a region within the promoter CpG island of *Wnt5a* as close as possible to regions previously studied by methyl-specific PCR (Mazieres *et al*, 2004; Ying *et al*, 2008). We also designed primers and a probe to amplify the genomic repeat sequence *Alu-C4* to normalise for DNA amount. This is a methylation-independent reaction and minimises amplification bias due to potential sample aneuploidy (Weisenberger *et al*, 2005). Primer and probe sequences were (*Wnt5a* forward) 5′-TTT AGG TTC GGG CGT TAG TGT TC -3′ (*Wnt5a* reverse) 5′-TCA AAA ACA AAA CTA CGA AAT CCT CC-3′ (*Wnt5a* probe) 6FAM-5′-TTT AGT TTC GGT TTA TTG CGT TCG TCG GA-3′-MGBNFQ; (ALU-C4 forward) 5′-GGT TAG GTA TAG TGG TTT ATA TTT GTA ATT TTA GTA-3′ (ALU-C4 reverse) 5′-ATT AAC TAA ACT AAT CTT AAA CTC CTA ACC TCA-3′ (ALU-C4 probe) 6FAM-5′-CCT ACC TTA ACC TCC C-3′-MGBNFQ.

Samples were analysed in 96-well plates on an ABI 7500 RT–PCR thermocycler as previously described (Weisenberger *et al*, 2005). Methylation was calculated as follows (Weisenberger *et al*, 2006): PMR=(gene/Alu-C4)_sample_/(gene/Alu-C4)_CpGenome_ × 100%. CpGenome DNA (Millipore, Billerica, MA, USA) was exogenously methylated lymphocyte DNA that was amplified on each plate to define a 100% reference signal for each gene. Receiver operator curves were generated from a subset of 100 tumour-matched normal pairs from Ontario to produce a tumour-specific methylation cutoff (PMR=10). This cutoff was used to define positive methylation and is a conservative value for transcriptional silencing of other genes (Ogino *et al*, 2006). For cases with multiple DNA samples due to synchronous baseline tumours (ON: *n*=34; NFLD: *n*=16), PMR values were averaged.

Validation was performed on randomly selected samples to account for interplate variation. Per cent methylated reference readings during validation tended to fluctuate <10% and reassignment of methylation status was exceptionally rare. During screening and validation, samples with an Alu-C4 threshold cycle above 22.0 were considered poor quality, retreated with sodium bisulphite, and subsequently reanalysed to ensure robust amplification. Samples that remained poor quality were removed from the analysis (ON: *n*=16; NFLD: *n*=34).

### Statistical analysis

The selectivity of Wnt5a methylation for tumour tissue over matched normal tissue was assessed using McNemar's *χ*^2^-test. All univariate clinicopathological associations were assessed by Fisher's exact test. Multivariate modelling of MSI status was conducted using unconditional logistic regression adjusted for age, sex, and tumour location. Unadjusted associations to recurrence-free survival status were assessed using Kaplan–Meier analysis with the log-rank test. Multivariate associations to recurrence-free survival status were conducted using a Cox regression model adjusted for age, sex, stage, and grade, with and without MSI status. Time-to-event was calculated from the date of diagnosis to the date of first recurrence/death or censored at the date of last contact. A two-sided *P*<0.05 was considered statistically significant. Bonferroni corrections for multiple comparisons were incorporated, where appropriate. Analysis was performed using SPSS version 16.0 (SPSS Inc., Chicago, IL, USA).

## Results

### Methylation analysis

We quantified promoter methylation levels of the *Wnt5a* gene in 545 colorectal carcinomas from Ontario and 687 colorectal carcinomas from Newfoundland. *Wnt5a* was methylated in 107 (19.6%) cases in Ontario and 125 (18.2%) cases in Newfoundland (Table 1). To verify that these methylation events were specific to tumour tissue, we requantified *Wnt5a* methylation levels in 53 tumour samples in parallel with their matched normal colonic mucosa samples (oversampling was conducted for positive tumour methylation to increase the number of informative samples). Wnt5a was methylated in 27 of these tumours (17 MSI, 10 MSS) and no methylation was found in matched normal tissue.

### Methylation and clinicopathological features

We examined associations between *Wnt5a* methylation status and patient clinicopathological features in both populations. In Ontario, *Wnt5a* methylation was much more common in MSI tumours than in MSS tumours (odds ratio (OR)=6.3, 95% confidence interval (CI)=3.9–10.4, *P*<10^−12^) (Table 2). This relationship was reflected in associations between *Wnt5a* methylation and MSI-associated tumour characteristics, including immunohistochemical deficiency of at least one mismatch repair protein (OR=7.2, 95% CI=4.3–12.2, *P*<10^−12^), *MLH1* methylation (OR=14.9, 95% CI=7.8–28.5, *P*<10^−16^), proximal location (OR=3.6, 95% CI=2.3–5.6, *P*<10^−7^), mucinous histological type (OR=2.0, 95% CI=1.1–3.7, *P*=0.03), and female gender (OR=2.5, 95% CI=1.6–3.8, *P*<10^−4^) (Table 2). There was, however, no association between *Wnt5a* methylation and germline mutations in mismatch repair gene, which cause the inherited MSI tumours found in Lynch syndrome (OR=0.3, 95% CI=0.1–1.6, *P*=0.24). The absence of this association to Lynch-associated MSI tumours was maintained after adjustment for age (data not shown), indicating the absence of confounding effects due to age-associated methylation. Indeed, *Wnt5a* methylation was not associated with age ⩾50 years compared with age <50 years (OR=1.5, 95% CI=0.7–3.1, *P*=0.39). *Wnt5a* methylation was also strongly associated with *BRAF* V600E mutation (OR=12.3, 95% CI=6.9–21.7, *P*<10^−17^), a common alteration in MSI and CIMP, and this association remained after adjustment for MSI status (OR=7.9, 95% CI=4.2–14.7, *P*<10^−10^).

An identical analysis was conducted in our validation cohort from Newfoundland. *Wnt5a* methylation was similarly associated with MSI tumours (OR=5.0, 95% CI=3.0–8.4, *P*<10^−18^) and the same set of MSI-associated characteristics: MMR IHC deficiency (OR=6.2, 95% CI=6.6–10.7, *P*<10^−9^), *MLH1* methylation (OR=36.7, 95% CI=10.3–130.4, *P*<10^−10^), proximal location (OR=3.2, 95% CI=2.1–4.9, *P*<10^−7^), mucinous histological type (OR=1.9, 95% CI=1.1–3.2, *P*=0.02), and female gender (OR=1.6, 95% CI=1.1–2.3, *P*=0.03) (Table 2). *Wnt5a* methylation remained unassociated with age ⩾50 years (OR=1.7, 95% CI=0.9–3.4, *P*=0.13), but showed a borderline inverse association with germline MMR gene mutation – no methylated cases carried these mutations, whereas 17 unmethylated cases (3%) carried these mutations (*P*=0.05). *Wnt5a* methylation also remained strongly associated with *BRAF* V600E before (OR=25.9, 95% CI=14.4–46.3, *P*<10^−33^) and after (OR =20.9, 95% CI=11.4–38.5, *P*<10^−21^) adjustment for MSI status.

Methylation status of *Wnt5a* was weakly associated or not associated with several other clinicopathological characteristics in one population, including: pathological N and M substages, local invasion, patient history of irritable bowel syndrome, and patient history of inflammatory bowel disease (Supplementary Table 1). Notably, however, *Wnt5a* methylation increased with grade in both populations and trended towards an association with increasing tumour stage and, more specifically, pathological T substage in Newfoundland (Supplementary Table 1).

### Methylation and multivariate MSI

We constructed a multivariate MSI model to adjust for the possible confounders of age, sex, and tumour location (Table 3). These covariates were each individually associated with MSI status in both populations (data not shown). After adjustment for these factors, *Wnt5a* methylation remained strongly associated with MSI in both Ontario (OR=4.2, 95% CI=2.4–7.5, *P*<10^−6^) and Newfoundland (OR=3.8, 95% CI=2.2–6.6, *P*<10^−5^).

### Methylation and recurrence-free survival

To assess the prognostic role of *Wnt5a* methylation, we conducted recurrence-free survival analysis. In Ontario, there were 120 recurrences and/or deaths due to CRC with a mean interval of 30 months from diagnosis. The mean follow-up time among remaining cases was 97 months. *Wnt5a* methylation was not significantly associated with outcome in unadjusted analysis (HR=0.7, 95% CI=0.4–1.1, *P*=0.13) or after adjustment for age, sex, stage, and grade (HR=0.8, 95% CI=0.5–1.4, *P*=0.42).

A similar analysis was conducted in Newfoundland, where there were 244 recurrences and/or deaths with a mean interval of 26 months from diagnosis. The mean follow-up time among remaining cases was 65 months. Again, *Wnt5a* methylation was not associated with outcome in unadjusted analysis (HR=1.0, 95% CI=0.7–1.4, *P*=0.83) or after adjustment for age, sex, stage, and grade (HR=0.8, 95% CI=0.6–1.2, *P*=0.28).

To assess the potential effects of MSI status on these relationships, we verified that MSI was a prognosticator of favourable outcome in both Ontario (HR=0.3, 95% CI=0.2–0.7, *P*=0.002) and Newfoundland (HR=0.5, 95% CI=0.3–0.8, *P*=0.007) and subsequently added an MSI covariate to the multivariate survival model. After this additional adjustment for MSI status, there was clearly no association between *Wnt5a* methylation and patient outcome in either Ontario (HR=1.0, 95% CI=0.6–1.7, *P*=0.96) or Newfoundland (HR=0.9, 95% CI=0.6–1.3, *P*=0.53).

## Discussion

Wnt5a is emerging as an important functional and prognostic contributor in several cancers and is commonly silenced by promoter hypermethylation. In CRC, DNA methylation has a vital role in defining prognostic subtypes, and aberrant Wnt signalling is critical for carcinogenesis; however, the prevalence of *Wnt5a* methylation in CRC and its distribution among tumour subtypes is poorly understood. In an effort to determine the frequency of this event between CRC subtypes and its potential prognostic role, we examined *Wnt5a* promoter methylation levels in two distinct MSI-stratified Canadian populations: Ontario, a heterogeneous population with a moderate incidence of CRC, and Newfoundland, a founder population with a high incidence of CRC, particularly within families (Woods *et al*, 2005). Despite these differences, Wnt5a methylation was concordantly observed in approximately 20% of tumours from both population. This is a lower methylation frequency than previously reported in two small CRC series from East Asia (Ying *et al*, 2008; Ahn *et al*, 2011). *Wnt5a* methylation was absent from a subset of matched normal mucosa, verifying that this event is tumour specific.

Importantly, this event was unequally distributed among MSI subtypes; *Wnt5a* methylation was much more common in MSI tumours than in MSS tumours in both populations and these associations were independent of the combined effects of patient age, sex, and tumour location. Furthermore, *Wnt5a* methylation was strongly associated with *MLH1* methylation, the primary cause of sporadic MSI, but not associated with germline MMR gene mutations, which cause the inherited MSI tumours found in Lynch Syndrome. Consequently, *Wnt5a* appears to be preferentially methylated among sporadic MSI tumours compared with HNPCC-associated MSI tumours.

*Wnt5a* methylation was also strongly associated with the oncogenic BRAF V600E mutation, a surrogate marker of CIMP high (Ogino and Goel, 2008) that correlates with a distinct subset of CIMP-associated methylation markers (Tanaka *et al*, 2010). BRAF V600E is an independent prognosticator of poor outcome in CRC and seems to be important confounder to the prognostic roles of CIMP (Kim *et al*, 2009; Ogino *et al*, 2009). Combined status of BRAF V600E, CIMP, and MSI has been proposed to more accurately stratify prognostic groups in CRC (Kim *et al*, 2009; Ogino *et al*, 2009; Dahlin *et al*, 2010). The strong and independent associations of *Wnt5a* methylation with both MSI and BRAF V600E suggest that this event may be a marker of MSI/CIMP-high tumours, which seem to be a particularly favourable CRC subset in these studies. Since promoter methylation of Wnt5a induces transcriptional silencing in CRC cells (Ying *et al*, 2008), our results suggest that MSI and/or CIMP-high tumours may experience preferential repression of non-canonical Wnt activity. These findings extend our previous results that suggested MSS tumours may be preferentially permissive to non-canonical Wnt stimulation due to the methylation status of Wnt antagonist genes (Rawson *et al*, 2011). Consequently, multiple epigenetic events may promote differential non-canonical Wnt stimulation between CRC subtypes.

In addition to its effects on non-canonical Wnt activity, Wnt5a is also known to inhibit *β*-catenin accumulation in CRC cells (Ying *et al*, 2008; Yuzugullu *et al*, 2009), suggesting that MSI and/or CIMP-high tumours may also experience preferential activation of canonical Wnt activity. This may be particularly important during development of MSI and CIMP tumours due to the relative absence of *APC* mutation as a means to induce cytoplasmic *β*-catenin accumulation in these tumours (Thorstensen *et al*, 2005; Samowitz *et al*, 2007; Albuquerque *et al*, 2010). Promoter methylation of the *DKK1* gene, which encodes a pivotal antagonist of the canonical Wnt pathway, has already been strongly associated with MSI (Rawson *et al*, 2011), begging the question whether canonical Wnt activity may be primarily driven by upstream epigenetic changes rather than downstream genetic changes in this subset of CRCs. Further investigation of the epigenetic status of other Wnt regulatory genes between CRC subtypes will be necessary to scrutinise this hypothesis.

Despite the attractive downstream effects of Wnt5a on cell signaling, the impact of subtype-associated Wnt5a methylation on subtype-specific CRC outcome is unclear. By offering both tumour-suppressive and oncogenic effects, the opposing regulatory functions of Wnt5a on canonical and non-canonical Wnt pathways may obscure the prognostic role of *Wnt5a* methylation in our analysis and help explain the conflicting results obtained in other cancers. Indeed, it has been proposed that these discordant functions of Wnt5a may affect CRC prognosis in a time-dependent manner, whereby early Wnt5a expression exerts a favourable effect by inhibiting tumour growth through canonical Wnt suppression, whereby late expression of *Wnt5a* exerts a deleterious effect by promoting tumour invasion through non-canonical Wnt activation (Wang, 2009). This model is harmonious with the identification of Wnt5a expression as a marker of favourable outcome in early CRC (Dejmek *et al*, 2005). Since methylation was well represented in both early and late stage tumours within our populations, this may help explain the absence of a significant association to recurrence-free survival despite the strong association to MSI. In addition, the prognostic relevance of *Wnt5a* methylation may be confounded by the status of downstream canonical and non-canonical Wnt mediators that may influence the signalling response to Wnt5a loss. Importantly, these effects may also be related to tumour subtype since alterations in canonical Wnt components seem to vary with MSI status (Thorstensen *et al*, 2005; Ortega *et al*, 2008; Albuquerque *et al*, 2010), although subtype specificity in non-canonical Wnt pathway components has not been explored.

To our knowledge, this is the first study to identify an association between *Wnt5a* methylation and cancer subtype. These findings extend the perspective that epigenetic events may promote differential Wnt signalling between tumour subtypes. Determining the exact functional and prognostic significant of these events may rely on investigating the collective genetic and epigenetic status of other Wnt mediators in the context of tumour subtype.

## Figures and Tables

**Table 1:** Distribution of clinicopathological features, including *Wnt5a* methylation in primary colorectal carcinomas from Ontario and Newfoundland

	**No. of cases (%)**	
	**Ontario**	**Newfoundland**
Cases of primary colorectal carcinoma	545	687
Mean age (s.d.)	60.7 (8.8)	61.3 (9.4)
White ethnicity	545 (100)	687 (100)
		
*Age*		
<50	61 (11.2)	83 (12.1)
50+	484 (88.8)	604 (87.9)
		
*Sex*		
Female	261 (47.9)	264 (38.4)
Male	284 (52.1)	423 (61.6)
		
*Tumour location* a		
Proximal	208 (38.2)	287 (41.8)
Distal	329 (60.4)	390 (56.8)
Unavailable	8 (1.5)	10 (1.5)
		
*TNM stage*		
1	95 (17.4)	101 (14.7)
2	211 (38.7)	235 (34.2)
3	165 (30.3)	206 (30.0)
4	30 (5.5)	144 (21.0)
Unavailable	44 (8.1)	1 (0.1)
		
*Histological grade*		
Low	43 (7.9)	99 (14.4)
Moderate	390 (71.6)	504 (73.4)
High	63 (11.6)	71 (10.3)
Unavailable	49 (9.0)	13 (1.9)
		
*Histological type* b		
Non-mucinous	444 (81.5)	601 (87.5)
Mucinous	62 (11.4)	86 (12.5)
Unavailable	39 (7.2)	0 (0.0)
		
*MSI status*		
Stable	385 (70.6)	585 (85.2)
Low	68 (12.5)	27 (3.9)
High	87 (16.0)	68 (9.9)
Unavailable	5 (0.9)	7 (1.0)
		
*MMR germline mutation*		
No	178 (32.7)	670 (97.5)
Yes	15 (2.8)	17 (2.5)
Unavailable	352 (64.6)	
		
*MMR protein status*		
Intact	444 (81.5)	605 (88.1)
Deficient	76 (13.9)	61 (8.9)
Unavailable	25 (4.6)	21 (3.1)
		
*MLH1 methylation*		
No	492 (90.3)	410 (59.7)
Yes	52 (9.5)	19 (2.8)
Untested	1 (0.2)	258 (37.6)
		
*BRAF V600E mutation*		
No	463 (85.0)	568 (82.7)
Yes	63 (11.6)	77 (11.2)
Unavailable	19 (2.2)	42 (6.1)
		
*Wnt5a methylation*		
Unmethylated	438 (80.4)	562 (81.8)
Methylated	107 (19.6)	125 (18.2)

Abbreviations: BRAF=v-raf murine sarcoma viral oncogene homolog B1; MLH1=mutL homolog 1; MMR=mismatch repair; MSI=microsatellite instability; TNM=tumour node metastasis.

a Proximal tumour location includes lesions up to and including the splenic flexure.

b Mucinous histology includes the presence of any mucin within the tumour stroma.

**Table 2:** Associations between *Wnt5a* methylation and age, sex, MSI status, MSI-associated features, and *BRAF* V600E status

	**Ontario**				**Newfoundland**			
	**Unmethylated (%)**	**Methylated (%)**	**OR (95% CI)**	***P***	**Unmethylated (%)**	**Methylated (%)**	**OR (95% CI)**	***P***
*Age*								
<50	52 (11.9)	9 (8.4)	1.0	0.39	73 (13.0)	10 (8.0)	1.0	0.13
50+	386 (88.1)	98 (91.6)	1.5 (0.7, 3.1)		489 (87.0)	115 (92.0)	1.7 (0.9, 3.4)	
								
*Sex*								
M	247 (56.4)	37 (34.6)	1.0	5.90 × 10^−5^^*^	357 (63.5)	66 (52.8)	1.0	0.03
F	191 (43.6)	70 (65.4)	2.5 (1.6, 3.8)		205 (36.5)	59 (47.2)	1.6 (1.1, 2.3)	
								
*Location*								
Distal	291 (67.2)	38 (36.5)	1.0	2.21 × 10^−8^^*^	347 (62.9)	43 (34.4)	1.0	1.20 × 10^−8^^*^
Proximal	142 (32.8)	66 (63.5)	3.6 (2.3, 5.6)		205 (37.1)	82 (65.6)	3.2 (2.1, 4.9)	
								
*Histological type*								
Non-mucinous	365 (89.5)	79 (80.6)	1.0	0.03	500 (89.0)	101 (80.8)	1.0	0.02
Mucinous	43 (10.5)	19 (19.4)	2.0 (1.1, 3.7)		62 (11.0)	24 (19.2)	1.9 (1.1, 3.2)	
								
*MSI status*								
MSS	390 (90.1)	63 (58.9)	1.0	7.56 × 10^−13^^*^	521 (93.5)	93 (74.4)	1.0	7.24 × 10^−19^^*^
MSI	43 (9.9)	44 (41.1)	6.3 (3.9, 10.4)		36 (6.5)	32 (25.6)	5.0 (3.0, 8.4)	
								
*MMR protein status*								
Intact	382 (91.6)	62 (60.2)	1.0	2.83 × 10^−13^^*^	513 (94.7)	92 (74.2)	1.0	2.81 × 10^−10^^*^
Deficient	35 (8.4)	41 (39.8)	7.2 (4.3, 12.2)		29 (5.3)	32 (25.8)	6.2 (3.6, 10.7)	
								
*MMR germline mutation*								
No	123 (90.4)	55 (96.5)	1.0	0.24	545 (97.0)	125 (100.0)	1.0	0.05
Yes	13 (9.6)	2 (3.5)	0.3 (0.1, 1.6)		17 (3.0)	0 (0.0)	—	
								
*MLH1 methylation*								
No	492 (90.4)	70 (65.4)	1.0	1.08 × 10^−17^^*^	358 (99.2)	52 (76.5)	1.0	2.06 × 10^−11^^*^
Yes	52 (9.6)	37 (34.6)	14.9 (7.8, 28.5)		3 (0.8)	16 (23.5)	36.7 (10.3, 130.4)	
								
*BRAF V600E*								
No	405 (94.6)	60 (58.8)	1.0	2.61 × 10^−18^^*^	508 (96.4)	60 (50.8)	1.0	5.78 × 10^−33^^*^
Yes	23 (5.4)	42 (41.2)	12.3 (6.9, 21.7)		19 (3.6)	58 (49.2)	25.9 (14.4, 46.3)	

Abbreviations: BRAF=v-raf murine sarcoma viral oncogene homolog B1; CI=confidence interval; MMR=mismatch repair; MSI=microsatellite instability; MSS=microsatellite stable; OR=odds ratio; TNM=tumour node metastasis.

^*^Significant *P*-value after Bonferroni correction (*P*<5.55 × 10^−3^).

— OR cannot be computed due to zero cell count.

**Table 3:** Multivariate MSI analysis adjusted for age, sex, tumour location, and *Wnt5a* methylation status

	**Ontario**			**Newfoundland**		
**Variable**	**Univariate *P***	**Multivariate *P***	**Multivariate OR (95% CI)**	**Univariate *P***	**Multivariate *P***	**Multivariate OR (95% CI)**
Age	0.95	0.16	0.98 (0.95, 1.01)	0.06	0.002	0.96 (0.94, 0.99)
Female Sex	0.003	0.27	1.36 (0.79, 2.35)	0.05	0.31	1.33 (0.77, 2.29)
Proximal Location	4.37 × 10^−15^	7.82 × 10^−12^	8.41 (4.57, 15.47)	1.31 × 10^−12^	7.74 × 10^−8^	6.21 (3.19, 12.09)
Wnt5a Meth	7.56 × 10^−15^	6.94 × 10^−7^	4.22 (2.39, 7.45)	7.24 × 10^−9^	3.94 × 10^−6^	3.78 (2.15, 6.64)

Abbreviations: CI=confidence interval; MSI=microsatellite instability; OR=odds ratio.
