# Supplementary material for: Promoter methylation of Wnt5a is associated with microsatellite instability and BRAF V600E mutation in two large populations of colorectal cancer patients
Source: Br J Cancer. 2011 May 17;104(12):1906–12. doi: 10.1038/bjc.2011.165 (PMC3111198; doi:10.1038/bjc.2011.165)
Supplement: Supplementary Table 1 [file bjc2011165x1.doc]

**Supp. Table 1.** Associations between *Wnt5a* methylation and other clinicopathological variables.

|  | **Ontario** | | | | |  | **Newfoundland** | | | | |
| --- | --- | --- | --- | --- | --- | --- | --- | --- | --- | --- | --- |
|  | **Total**  **(%)** | **Unmethylated**  **(%)** | **Methylated**  **(%)** | **OR (95% CI)** | **P** |  | **Total**  **(%)** | **Unmethylated**  **(%)** | **Methylation**  **(%)** | **OR (95% CI)** | **P** |
| **Stage** |  |  |  |  |  |  |  |  |  |  |  |
| 1 | 95 (17.4) | 79 (19.7) | 16 (16.0) | 1.0 | 0.44 |  | 101 (14.7) | 87 (15.5) | 14 (11.2) | 1.0 | 0.07 |
| 2 | 211 (38.7) | 162 (40.4) | 49 (49.0) | 1.5 (0.8, 2.8) |  |  | 235 (34.2) | 194 (34.6) | 41 (32.8) | 1.3 (0.7, 2.5) |  |
| 3 | 165 (30.3) | 134 (33.4) | 31 (31.0) | 1.1 (0.6, 2.2) |  |  | 206 (30.0) | 173 (30.8) | 33 (26.4) | 1.2 (0.6, 2.3) |  |
| 4 | 30 (5.5) | 26 (6.5) | 4 (4.0) | 0.8 (0.2, 2.5) |  |  | 144 (21.0) | 107 (19.1) | 37 (29.6) | 2.2 (1.1, 4.2) |  |
| **pT** |  |  |  |  |  |  |  |  |  |  |  |
| 1 | 40 (7.3) | 34 (8.1) | 6 (5.9) | 1.0 | 0.18 |  | 32 (4.7) | 26 (4.6) | 2 (1.6) | 1.0 | 0.03 |
| 2 | 101 (18.5) | 86 (20.5) | 15 (14.9) | 1.0 (0.4, 2.8) |  |  | 114 (16.6) | 96 (17.1) | 18 (14.4) | 2.4 (0.5, 11.2) |  |
| 3 | 349 (64.0) | 279 (66.4) | 70 (69.3) | 1.4 (0.6, 3.5) |  |  | 467 (68.0) | 385 (68.5) | 82 (65.6) | 2.8 (0.6, 11.9) |  |
| 4 | 31 (5.7) | 21 (5.0) | 10 (9.9) | 2.7 (0.9, 8.5) |  |  | 74 (10.8) | 51 (9.1) | 23 (18.4) | 5.9 (1.3, 26.8) |  |
| **pN** |  |  |  |  |  |  |  |  |  |  |  |
| 0 | 315 (57.8) | 248 (62.0) | 67 (67.0) | 1.0 | 0.25 |  | 354 (51.5) | 294 (53.6) | 60 (48.8) | 1.0 | 0.08 |
| 1 | 131 (24.0) | 111 (27.8) | 20 (20.0) | 0.7 (0.4, 1.2) |  |  | 186 (27.1) | 156 (28.5) | 30 (24.4) | 0.9 (0.6, 1.5) |  |
| 2 | 54 (9.9) | 41 (10.3) | 13 (13.0) | 1.2 (0.6, 2.3) |  |  | 131 (19.1) | 98 (17.9) | 33 (26.8) | 1.7 (1.0, 2.7) |  |
| **pM** |  |  |  |  |  |  |  |  |  |  |  |
| 0 | 491 (90.1) | 394 (93.8) | 97 (96.0) | 1.0 | 0.48 |  | 543 (79.0) | 455 (81.0) | 88 (70.4) | 1.0 | 0.01 |
| 1 | 30 (5.5) | 26 (6.2) | 4 (4.0) | 0.6 (0.2, 1.8) |  |  | 144 (21.0) | 107 (19.0) | 37 (29.6) | 1.8 (1.2, 2.7) |  |
| **Grade** |  |  |  |  |  |  |  |  |  |  |  |
| Low | 43 (7.9) | 32 (8.0) | 11 (11.5) | 1.0 | 0.05 |  | 99 (14.4) | 86 (15.5) | 13 (10.8) | 1.0 | 6.23E × 10^-5^ |
| Moderate | 390 (71.6) | 323 (80.8) | 67 (69.8) | 0.6 (0.3, 1.3) |  |  | 504 (73.4) | 424 (76.5) | 80 (66.7) | 1.3 (0.7, 2.3) |  |
| High | 63 (11.6) | 45 (11.3) | 18 (18.8) | 1.2 (0.5, 2.8) |  |  | 71 (10.3) | 44 (7.9) | 27 (22.5) | 4.1 (1.9, 8.6) |  |
| **Local Invasion** |  |  |  |  |  |  |  |  |  |  |  |
| No | 57 (10.5) | 43 (33.6) | 14 (42.4) | 1.0 | 0.42 |  | 275 (40.0) | 228 (45.3) | 47 (42.3) | 1.0 | 0.60 |
| Yes | 104 (19.1) | 85 (66.4) | 19 (57.6) | 0.7 (0.3, 1.5) |  |  | 339 (49.3) | 275 (54.7) | 64 (57.7) | 1.1 (0.8, 1.7) |  |
| **History of Irritable Bowel Syndrome** | |  |  |  |  |  |  |  |  |  |  |
| No | 458 (84.0) | 363 (91.9) | 95 (92.2) | 1.0 | 1.00 |  | 587 (85.4) | 475 (94.4) | 112 (96.6) | 1.0 | 0.49 |
| Yes | 40 (7.3) | 32 (8.1) | 8 (7.8) | 1.0 (0.4, 2.1) |  |  | 32 (4.7) | 28 (5.6) | 4 (3.4) | 0.6 (0.2, 1.8) |  |
| **History of Inflammatory**  **Bowel Disease** | |  |  |  |  |  |  |  |  |  |  |
| No | 385 (70.6) | 307 (80.8) | 78 (78.8) | 1.0 | 0.61 |  | 508 (73.9) | 409 (79.0) | 99 (83.9) | 1.0 | 0.25 |
| Yes | 94 (17.2) | 73 (19.2) | 21 (21.2) | 1.1 (0.7, 2.0) |  |  | 128 (18.6) | 109 (21.0) | 19 (16.1) | 0.7 (0.4, 1.2) |  |
